# Incidental detection of retinoblastoma from accidental trauma in children

**DOI:** 10.1186/s12886-023-02819-2

**Published:** 2023-02-28

**Authors:** Yuxin Fang, Qiyan Li, Chengyue Zhang, Junyang Zhao

**Affiliations:** 1 Beijing Ophthalmology & Visual Sciences Key Lab, Beijing Tongren Eye Center, Beijing TongRen Hospital, Capital Medical University, Beijing, 100730 China; 2 Department of Ophthalmology, National Center for Children’s Health, Beijing Children’s Hospital, Capital Medical University, Beijing, 100045 China; 3 Pediatric Oncology Center, National Center for Children’s Health, Beijing Children’s Hospital, Capital Medical University, Beijing, 100045 China

**Keywords:** Retinoblastoma, Ocular trauma, Pediatrics, CT scan, Metastasis

## Abstract

**Background:**

To report a case series of patients who were diagnosed with retinoblastoma (RB), which was preceded by trauma, in a large multicenter cohort and to investigate the incidence, clinical characteristics, and causes of RB misdiagnosis.

**Methods:**

The medical records of consecutive patients with RB between 2006 and 2015 were retrospectively reviewed. Characteristics of trauma patients, including their age at initial trauma, site of trauma, sex, and RB laterality, were analyzed.

**Results:**

Among 3780 patients, 30 (0.8%) experienced systemic or ocular trauma prior to the detection of RB. The median age was 20.7 months, and the median follow-up time was 6 years. There were 2 eyes in stage A, 2 in stage B, 3 in stage C, 12 in stage D, and 15 in stage E. The remaining 2 eyes had extraocular RB. A total of 20 patients experienced ocular trauma, 9 patients experienced head trauma, and 1 patient experienced trauma in other body parts. RB was suspected or detected in 22 patients (73.3%) at the time of primary trauma occurrence, and 8 patients (26.7%) were misdiagnosed with RB during their first visit.

Among them, all experienced blunt ocular trauma, and enucleation was performed in 7 patients in which 1 patient died.

**Conclusions:**

Less than 1% of the patients experienced systemic or ocular trauma before RB was detected. The majority were unilateral and in advanced stages. Differential diagnoses that are not trauma-related must always be considered, and comprehensive examinations must be conducted before diagnostic and therapeutic intraocular procedures are initiated.

## Background

Ocular injuries account for 8–14% of total injuries in children and are the most significant cause of unilateral blindness in children [1]. The annual incidence of severe ocular trauma in children has been estimated to be around 8.85 to 15.2 per 100,000 [2–5]. Ocular trauma presentations include a wide range of signs and symptoms, including mild subconjunctival hemorrhage, traumatic cataracts, hyphema, vitreous hemorrhage, traumatic retinal detachment, and more. Non-compliance with ophthalmic examination and an unreliable history from children may potentially increase the difficulty of obtaining an accurate diagnosis, which may subsequently delay the initiation of treatment. The management of trauma patients is complex, especially in children, and timely surgery is needed. A comprehensive examination is necessary for children. In some cases, situations other than trauma-related ones must also be considered.

Retinoblastoma (RB) is the most common intraocular malignant tumor in children. The survival rate of children with RB has dramatically improved over the years. A high survival rate can only be achieved with an accurate diagnosis and effective treatment plan [6, 7]. Leukocoria is the most common sign of RB [8–10]. Ancillary testing, including B-scan ultrasound and computerized tomography, is somewhat mandatory when RB is suspected. However, RB may also present with unusual signs, such as vitreous hemorrhage or inflammation, leading to misdiagnosis and inappropriate treatment. Evidently, these misleading or masquerading signs, coupled with a definite trauma history, will inevitably make it more difficult to accurately diagnose RB. In our clinical practice, although patients with RB who first present with trauma are relatively not common, the recognition of such situation remains critical for the following reasons: signs and symptoms are often easy to ignore and delayed RB management is associated with a high risk of extraocular, central nervous system, bone marrow, or other metastatic diseases.

In this article, we report the primary trauma type, initial diagnosis/misdiagnosis, clinical presentation, management, and outcomes of patients who experienced trauma before they were diagnosed with RB. We aimed to investigate the incidence, clinical characteristics, and more significantly, the causes of misdiagnosis contribute to the consideration of RB as a differential diagnosis in such cases.

## Materials and methods

We retrospectively reviewed the medical records of consecutive patients with RB between 2006 and 2015 at 29 Chinese treatment centers over a 10-year period. Patients who had experienced trauma before the diagnosis of RB were selected and studied in further detail. This study adhered to the principles of the Declaration of Helsinki. Ethics Committee approval was obtained from Beijing Children’s Hospital. Signed informed consent was obtained from all of the children’s parents.

The collected patient data included age at initial trauma occurrence, sex (male, female), RB laterality (unilateral, bilateral), and heredity (sporadic, familial). The primary clinical presentation, diagnosis, and management before RB was suspected were also analyzed. Additionally, the site of trauma was noted. Intraocular RB staging at the time of detection was applied based on the International Classification of Intraocular Retinoblastoma (IIRC).[11] The interval between trauma occurrence and RB diagnosis was also calculated. Histopathological details of the enucleated eye were noted. Treatment outcomes (alive/dead) were recorded. The development of orbital recurrence or distant metastasis was recorded during the follow-up period.

For the statistical analyses, we used a statistical software package (SPSS version 22.0 IBM-SPSS, Chicago, IL). We first described the distribution of the main parameters by calculating their median or mean and standard deviation. We compared the assessed parameters between the two groups using the Student’s t-test or Mann–Whitney U test. Frequencies were compared using the chi-square test. The enucleation-free survival was determined using the Kaplan–Meier method and compared using the log-rank test. A *P*-value of < 0.05 was considered to be statistically significant.

## Results

The medical records of 3,780 consecutive patients with RB from 2006 to 2015 were reviewed. Of these, 36 eyes of 30 patients (0.8%) experienced systemic or ocular trauma before RB was detected. There were 20 males and 10 females, with 24 patients having unilateral RB and 6 patients having bilateral RB. The patients also had no positive family history for RB. The median age during the first visit was 20.7 months, with a range of 4.9 to 134.3 months (mean: 33.6 months). According to the IIRC, 2 eyes belonged to the A group, 2 eyes belonged to the B group, 3 eyes belonged to the C group, 12 eyes belonged to the D group, and 15 eyes belonged to the E group. The other 2 eyes had extraocular RB. Of the 36 eyes, enucleation was performed in 25 eyes (69.4%), and local tumor control was achieved in 11 eyes (30.6%), including all eyes (7 eyes) in groups A, B, and C; 3 eyes in group D; and 1 eye in group E. During the last follow-up period, 2 patients (2/30,6.7%) were lost to follow-up, while the other 2 patients (6.7%) died of metastasis. The remaining 26 patients (86.7%) were able to proceed with their follow-up, and the median follow-up interval was 6.0 years (range, 2–12 years).

**Fig. 1:**
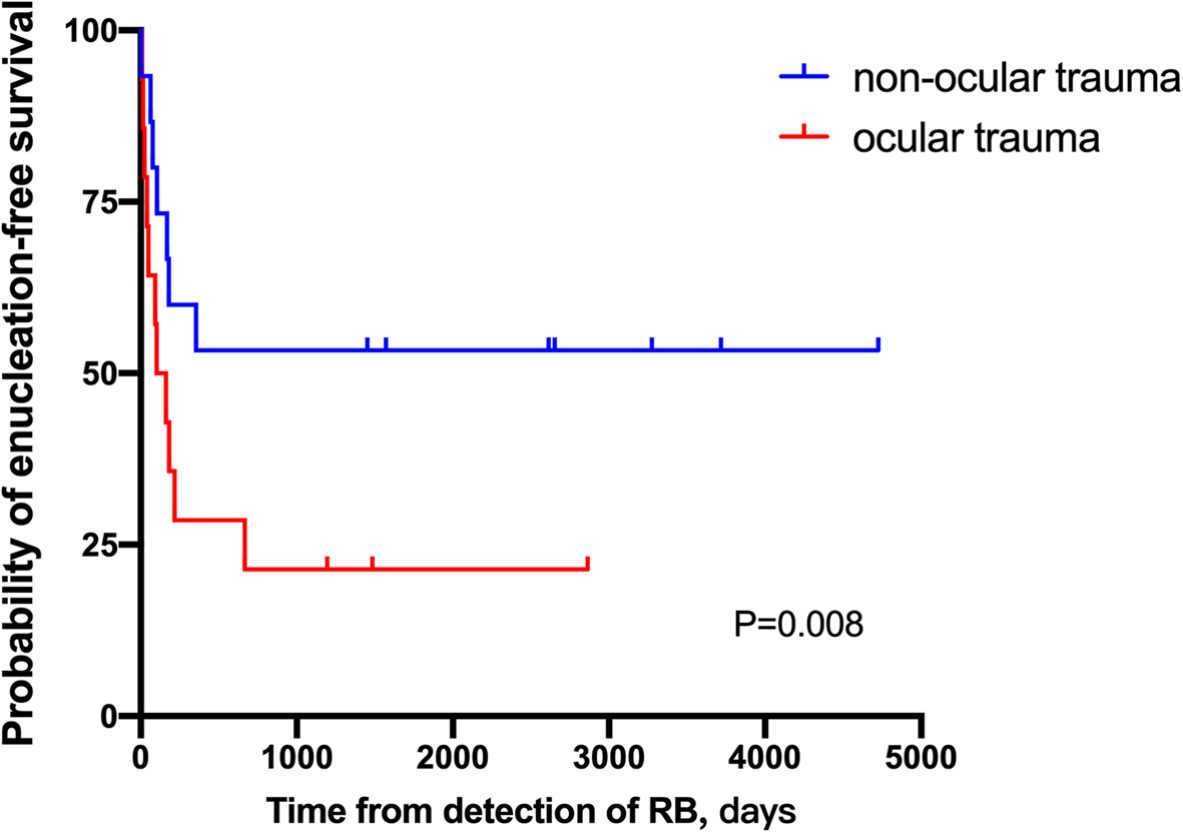
Kaplan–Meier survival curve and survival analysis showing enucleation-free survival in both ocular and non-ocular trauma groups

Based on the body area involving the trauma, 20 patients experienced ocular trauma, 9 patients experienced head trauma, and 1 patient experienced trauma in other body parts. The clinical characteristics and treatment outcomes based on the different body areas of the primary trauma are described in Table 1. The patients with head trauma and trauma involving other parts were grouped together (non-ocular) (10 patients). Notably, the patients with ocular trauma (mean rank = 18.2 months) were significantly older than those with non-ocular trauma (mean rank = 10.1 months) (U = 46, Z = -2.376, *P* = 0.017). However, RB was more likely to be suspected or detected during the first time of trauma event in patients with other body traumas (100%) than in those with ocular trauma (12 patients, 60%) (*P* = 0.029). Among the 10 patients with body trauma, 4 children presented with abnormal ocular signs, in which 3 eyes had conjunctival hyperemia and 1 eye showed leukocoria. The interval between trauma occurrence and RB diagnosis was seemingly longer in patients with ocular trauma, although statistical analysis did not show any significance (U = 74.5, Z = -1.127, *P* = 0.267). Even though there was no difference in the proportion of groups D and E between affected eyes with ocular trauma and other body trauma (*P* = 0.103), the prevalence of global salvage was much higher in eyes with primary head and other body trauma than in those with ocular trauma (53.3% vs. 14.3%; *P* = 0.025). Enucleation-free survival in both the ocular and non-ocular trauma groups is shown in Fig. 1. Kaplan–Meier survival analysis demonstrated ocular trauma groups had a significant lower enucleation-free survival compared with non-ocular trauma groups (*P* = 0.008). The median time to enucleation in eyes with ocular trauma was 41 days.

**Table 1:** The comparison of clinical characteristic and treatment outcomes between RB patients with ocular and non-ocular trauma

	Ocular trauma	Non-ocular trauma	*P* value
No.of patients (eyes)	20 (21)	10 (15)	
Sex			> 0.99
Male	13 (65%)	7 (66.7%)	
Female	7 (35%)	3 (33.3%)	
Median age at diagnosis (months)	38.0	12.1	0.017
Laterality			0.009
Bilateral	1	5	
Unilateral	19	5	
Suspicious/diagnosis of RB at the time of trauma	12 (60%)	10 (100%)	0.029
The interval time between trauma occurrence and RB diagnosis (days)	21.5 (0 to 1261)	10.5 (0–28)	0.267
IIRC stage of eyes			0.103*
A	0	2 (13.3%)	
B	1 (4.8%)	1 (6.7%)	
C	1 (4.8%)	2 (13.3%)	
D	5 (23.8%)	7 (46.7%)	
E	12 (57.1%)	3 (20%)	
Extraocular RB	2 (9.5%)	0	
Global salvage (eyes (%))	3 (14.3%)	8 (53.3%)	0.025
Status of patients at last visit			0.548**
Alive	17 (85%)	9 (90%)	
Died	2 (10%)	0	
Lost to follow up	1 (5%)	1 (10%)	

^*^: Comparison the proportion of group A, B and C stages and group D, E and extraocular stages between 2 trauma types. **: Comparison the proportion of alive and died groups between 2 t trauma types

RB, retinoblastoma; IIRC, International Classification of Intraocular Retinoblastoma

RB was suspected or detected in 22 patients (28 eyes, 73.3%) at the time of primary trauma occurrence. All 8 patients (8 eyes, 26.7%) who were misdiagnosed with RB at the first admission suffered from unilateral blunt ocular trauma (Table 2). There was no significant difference in the age and sex between patients with and without RB during the first visit (both *P* > 0.05). Among the 8 patients with a missed diagnosis of RB during the first time, the median age at diagnosis was 20.9 months (range, 5.7 to 150.9 months (mean: 36.5 months). The primary diagnosis included hyphemia/vitreous hemorrhage in 4 eyes (50%), cataract in 1 eye (12.5%), ocular foreign body in 1 eye (12.5%), secondary glaucoma in 1 eye (12.5%), and strabismus in 1 eye (12.5%). Ocular surgeries were performed in 5 eyes, in which pars plana vitrectomy (PPV) was completed in 3 eyes, and anterior chamber puncture and trabeculectomy were performed in the other 2 eyes. In the remaining 3 eyes, oral medications or topical eye drops were prescribed. All patients were diagnosed with unilateral RB in the traumatic eye. The median interval between trauma occurrence and RB diagnosis was 3.7 months (range: 0.5 to 41.4 months). All 8 eyes were in advanced stages of RB in D (6 eyes), E (1 eye), and extraocular (1 eye) staging, whereas 21 eyes (of 28, 75%) with early detection of RB were in the severe stage. The difference between the two groups did not reach statistical significance. Enucleation was performed in 7 eyes (87.5%) and local tumor control was achieved in 1 eye (12.5%); thus, the prevalence of global salvage was lower than that in the group with early detection of RB (35.7%), even though the statistical analysis was not significant. One patient (12.5%) was lost to follow-up and 1 patient who underwent PPV (12.5%) eventually died. The remaining 6 patients (75%) were able to continue with their follow-up.

**Table 2:** Clinical Characteristic of 8 Eyes with unsuspected RB at the first visit suffered from unilateral blunt ocular trauma

No	Age range at trauma occurrence	Sex	Eye	Primary Misdiagnosis	Surgical Intervention before RB suspected	Interval time between trauma occurrence and RB diagnosis (Days)	The reason finally suspected RB	IIRC stage of eyes	Patient status at last visit	Eye status at last visit	Follow-up time (years)
1	toddler	M	OS	Cataract	Eye drops	16	Referral	E	Alive	Enucleated	10
2	School age	M	OD	Hemorrhage (vitreous)	PPV	29	In surgery	D	Alive	Enucleated	7
3	toddler	M	OS	Hemorrhage (vitreous)	Oral medicine	60	Referral	E	Alive	Enucleated	6
4	toddler	M	OD	Strabismus	Eye drops	110	Referral	E	Alive	Stable	8
5	preschool	F	OD	Foreign body	AC puncture	116	Referral	E	Alive	Enucleated	7
6	Infant	F	OD	Hemorrhage (AC and vitreous)	Lensectomy + PPV + Silicon oil injection	194	IOP elevation and eye protrusion	E	Died	Enucleated	1
7	preschool	F	OD	Hemorrhage (vitreous)	PPV	677	IOP elevation after PPV	E	Lost	Enucleated	2
8	School age	M	OS	Glaucoma	Trabeculectomy, transscleral cyclophotocoagulation, glaucoma drainage device implantation	1260	Referral	E +	Alive	Enucleated	12

*RB* Retinoblastoma, *IIRC* International Classification of Intraocular Retinoblastoma, *AC* Anterior chamber, *PPV* Pars plana vitrectomy, *IOP* Intraocular pressure, *E +* Extraocular staging

Among the 30 patients, 2 (6.7%) with unilateral RB died due to metastasis. Both patients experienced ocular trauma. One 21-month-old child was diagnosed with RB wherein the tumor had invaded the orbit, which was confirmed by computerized tomography (CT) scans during the time of first admission. Evisceration was performed immediately, but the parents refused adjuvant chemotherapy. The patient died after 5 months. Another 6-month-old child who was primarily diagnosed with hyphemia and vitreous hemorrhage was treated with lensectomy-vitrectomy-silicon oil injection. One month after surgery, an elevated intraocular pressure was observed, and the affected eye was abnormally protruded. Hence, RB was highly suspected and finally diagnosed as stage E. Five months later, metastatic tumors of the optic nerve and brain were detected by re-examining a CT scan in a referral hospital. However, the child died 5 months after the affected eye was enucleated.

## Discussion

The most common initial ocular manifestation of RB reported by parents is leukocoria [8–10]. It is pertinent to rule out RB when leukocoria is noted in children. However, the presenting signs differ markedly depending on the extent of ocular involvement when RB is suspected. Thus, other less frequent presenting symptoms, which are often associated with late diagnosis, should also be considered, including painful glaucomatous eyes, hyphemia, hypopyon, and even proptosis due to posterior extraocular extension of the tumor in the orbit [8]. The diagnosis of RB after comprehensive examination is not difficult. However, it becomes challenging and often misleading when children encounter definite ocular or other body trauma when RB is less likely to be considered.

In our large cohort consisting of Chinese patients with RB, about 1 in 125 children experienced systemic or ocular trauma before RB was detected, which was comparable with a previous study reported by Chen et al. [12] who found 10 out of 793 RB patients (1.3%) had an isolated history of trauma. However, their population was only from a single tertiary center, and the number is much smaller than what is presented in this study. The median age during the first visit after trauma was 20 months, and the majority of the children (80%) were in advanced stages (IIRC group D, E, and extraocular RB). It is almost impossible for children who are only 20 months old to provide accurate details about their trauma or any symptoms related to visual impairment. A standard eye screening system has not been established in most regions of China, especially in the rural areas. The lack of routine eye screening for children and the poor parental education on the early signs of RB delay disease detection until the children are forced to visit the hospital due to trauma. In developing countries, pediatric eye injuries are also associated with poor education, poor economic conditions, and irresponsible child supervision [13]. Visual acuity can be significantly reduced in eyes with a rapidly growing tumor, which might be one of the reasons for injury in these children. Unilateral disease at the time of diagnosis in our study was seen in 80% of patients, which is much higher than previously reported hospital-based studies involving Asian patients with RB [9, 10] and also higher than each group that was graded based on their income level among 278 RB treatment centers worldwide [14]. As previous studies have shown, bilateral cases presented earlier than unilateral ones [10, 15], because of less advanced tumors were less frequently among unilateral cases as compared with bilateral cases. Early detection and accurate diagnosis prove to be even more challenging when the patient suffers from a definite trauma.

Among the 30 children with RB who visited the hospital because of trauma, not all were suspected of having or developing RB at the first time. Unexpectedly, RB was detected in all children who suffered from head and other body trauma, but in only 60% of the children with ocular trauma. The high detectability of RB in the pediatric clinic is mainly because brain CT scan is usually a routine examination for children who encounter brain injuries, especially when they fail to cooperate with other physical examinations. Besides that, it is reasonable for a pediatrician to refer the patients to an ophthalmologist when abnormal ocular signs are presented that do not fit the body trauma history. However, it is challenging to make an accurate diagnosis in children with a definite ocular trauma history. Common symptoms related to ocular trauma may mask the signs of RB. In addition, some “unnecessary physical examinations” can easily be omitted by ophthalmologists to save time, especially when emergency surgery is warranted.

The incidence of pediatric ocular trauma is higher in rural areas. However, there is still a lack of qualified ophthalmologists who can deal with the complex ocular condition in such local hospitals. Timely referral to a tertiary hospital and enhancement of the knowledge about RB for local doctors are crucial for not missing an RB diagnosis, which can be life-threatening. Even in hospitals specializing in ophthalmology, misdiagnosis can occur in some complicated cases. For example, in patient No. 6 (Table 2), the B-scan ultrasound was performed twice before PPV. In this case, it presented as a diffuse infiltrating RB, and no solid masses or calcification were detected.

There is wide variation in the global salvage and survival outcomes of children with RB among different studies [9, 10, 14–18]. In a large series of 1457 cases of RB from India in which advanced Group D/E tumors occupied 73% of intraocular tumors, the global salvage rate was 45%, and 5-year and 10-year survival estimates reached 90% and 89%, respectively [9]. In another hospital-based cohort from our group including 436 patients from 2013–2016, only 16% of eyes with RB were enucleated (IIRC class was not provided) and the mortality rate was 0.9% [10], indicating that with the advance of conservative therapy, enucleation was not the only option or the major treatment anymore. Another treatment center in China also showed that the enucleation rate constantly decreased over 10 years [18]. In the present study, patients with trauma as the first presentation had a relatively lower rate of global salvage (30.6%) and an average mortality rate (2/28, 7.1%) compared with the above-mentioned Asian data [9]. The delayed detection of RB in children with ocular trauma inevitably delays effective treatment and leads to much lower global salvage. Clinicians should be aware of the possibility that other than trauma-related diagnosis, especially for young children, comprehensive examination should always be performed before any intraocular procedure is performed. Carefully planned PPV and tumor resection can be a safe therapy without extraocular spread of tumors for those who are against standard treatments [19]. However, unplanned intraocular procedures should be avoided in these cases until the possibility of underlying RB is excluded [20, 21]. If surgery is performed in a child with RB, enucleation combined with chemotherapy, radiotherapy, or both without delay is strongly suggested, and most of the children can remain a good prognosis.

The potential limitations of this study include the following: In most cases, we needed detailed medical records of the first visit to a local hospital, which may have made it difficult to explore the other reasons for misdiagnosis. The low prevalence of patients with trauma at the first presentation remains limited; and the sample size may have also decreased the reliability of our statistical results.

## Conclusions

In conclusion, 0.8% of patients with RB experience systemic or ocular trauma as their first presentation. The median age during the first visit was 20.7 months, and the majority of tumors were unilateral and in advanced stages. All patients with body trauma were suspected of RB, however, there were 8 patients who experienced ocular trauma (27%) who were misdiagnosed. A lack of comprehensive examination, such as CT scans, evaluating trauma-related ocular signs masking the finding of RB, and shortage of qualified ophthalmologists in rural areas must be considered as major factors that contribute to the misdiagnosis of RB. Most children had a favorable prognosis, even though the global salvage rate was relatively low. We recommend that careful examinations for accidental ocular trauma in children be performed, and RB should always be ruled out in the first place.

## Data Availability

The datasets used and/or analyzed during the current study are available from the corresponding author upon reasonable request.

## Abbreviations

RB: Retinoblastoma (RB)
IIRC: International Classification of Intraocular Retinoblastoma
PPV: Pars Plana Vitrectomy
